# Non-Linear Hopped Chaos Parameters-Based Image Encryption Algorithm Using Histogram Equalization

**DOI:** 10.3390/e23050535

**Published:** 2021-04-27

**Authors:** Karim H. Moussa, Ahmed I. El Naggary, Heba G. Mohamed

**Affiliations:** 1 Electrical Department, College of Engineering, Horus University Egypt, New Damietta 34518, Egypt; khassan@horus.edu.eg; 2 Electrical Department, College of Engineering, King Marriott Institute of Engineering and Technology, Alexandria 23713, Egypt; anaggary@gmail.com; 3 Electrical Department, College of Engineering, Princess Nourah bint Abdulrahman University, Riyadh 11671, Saudi Arabia; 4 Electrical Department, College of Engineering, Alexandria Higher Institute of Engineering and Technology, Alexandria 21421, Egypt

**Keywords:** image encryption, information security, correlation, equalization, chaotic map, multimedia

## Abstract

Multimedia wireless communications have rapidly developed over the years. Accordingly, an increasing demand for more secured media transmission is required to protect multimedia contents. Image encryption schemes have been proposed over the years, but the most secure and reliable schemes are those based on chaotic maps, due to the intrinsic features in such kinds of multimedia contents regarding the pixels’ high correlation and data handling capabilities. The novel proposed encryption algorithm introduced in this article is based on a 3D hopping chaotic map instead of fixed chaotic logistic maps. The non-linearity behavior of the proposed algorithm, in terms of both position permutation and value transformation, results in a more secured encryption algorithm due to its non-convergence, non-periodicity, and sensitivity to the applied initial conditions. Several statistical and analytical tests such as entropy, correlation, key sensitivity, key space, peak signal-to-noise ratio, noise attacks, number of pixels changing rate (NPCR), unified average change intensity randomness (UACI), and others tests were applied to measure the strength of the proposed encryption scheme. The obtained results prove that the proposed scheme is very robust against different cryptography attacks compared to similar encryption schemes.

## 1. Introduction

Multimedia data such as text, audio, video, and image play a very important role in information security. One of the most important types of multimedia content is digital images due to their military applications, authentication of biometrics, medical science, and personal albums. In order to protect privacy and maintain the security of private images against unauthorized use or vulnerable attacks while passing through a public network, we need a trustable image encryption process. Many encryption schemes have been proposed, standardized, and widely adopted since the 1970s. These encryption schemes can vary between data encryption standard (DES) and advanced encryption standard (AES) techniques [1,2]. In 1963, Edward Lorenz applied chaos theory in computer systems [3]. Afterward, the cryptography schemes based on chaos theory were the primary choice for most cryptographers when proposing new encryption algorithms. Logistic map-based algorithms together with higher dimensional chaos functions lead to more secure encryption schemes against cryptanalytic attacks [4,5,6,7,8,9].

Recently, many low-dimensional chaotic systems have been developed [10,11,12]. These researchers proposed an encryption scheme with good chaos performance. Although these systems have low complexity, they are based on a fixed chaotic map which results in these low-dimensional systems becoming vulnerable to brute force attacks. Some encryption algorithms depending on logistic maps have been proposed in [13,14,15,16,17,18,19,20,21]. The digital image encryption schemes are mainly based on two processes, namely, position permutation, value transformation, or a combination of both processes. Position permutation is simply executed by fixing the pixel values and permuting the image position. On the other side, value transformation is accomplished by fixing the image position and assigning new values for the pixels. Due to its applicability and simplicity in implementation, the position permutation process is considered a primitive operation in most image encryption schemes. The encryption algorithms based on permutation-only processes show poor resistance against cipher text-only attacks and/or known/chosen-plaintext attacks and are only used in moderate or low-level security applications. The main purpose of the value transformation technique is to establish linear independency relations among several variables. Such operations can be accomplished simply through an XOR operation. The main advantage of the value transformation process is the non-reversibility manner, i.e., to reverse the value transformation operation we need the two arguments’ initial values used to create such a process, which is impossible to achieve.

In order to maintain the optimal security performance, several researchers proposed encryption schemes based on both processes, starting with position permutation, then applying value transformation. Most of the proposed algorithms to generate new pixel value during the value transformation process were depending on a fixed 3D chaotic map. To further increase the security of such image encryption schemes, we suggest a new encryption cryptosystem to generate a logistic parameter hopped 3D chaotic map that is used to generate the new pixel values during the value transformation process. We applied our proposed digital image encryption scheme to previously analyzed well-known images to compare our tests results with previous encryption schemes. The obtained results for our encryption scheme showed better performance results compared to other encryption schemes based on a fixed 3D chaotic map in terms of several types of attacks.

The rest of the article is organized as follows: Related image encryption schemes depending on 3D chaotic maps are briefly covered in Section 2; Section 3 explains the proposed image encryption cryptosystem that depends on a logistic parameter hopped 3D chaotic; Statistical tests used to evaluate the performance of our encryption scheme and simulation results are presented in Section 4; And, finally, Section 5 concludes the proposed algorithm.

## 2. Related Work

In different encryption schemes, a variety of strategies and different chaotic algorithms are adopted. Xiaoling Huang et al. [22] offered an encryption algorithm depending on the permutation–diffusion operation. The chaotic map output was revised through a middle parameter influenced by secret keys yielding to a temporal delay. Xu, L et al. [23] introduced a bit-level image encryption algorithm depending on piecewise linear chaotic maps (PWLCM). The authors transformed the plain image into two identical binary sequences. The two sequences generated were diffused mutually through a new diffusion strategy. Finally, they applied bits permutation through swapping the binary sequences by means of the chaotic map.

El-khamy, S.E. et al. [24] proposed a new chaotic image encryption algorithm depending on permutation and substitution in the Fourier domain. The authors achieved a large degree of randomization by applying a Fractional Fourier transform. Baker map, together with a generated key depending on a modified logistic map, was used for the permutation process yielding to an increase in the space of the encryption key. Dongdong Lin et al. [25] offered an image encryption cryptosystem based on information entropy. The authors evaluated the security metric validity and security properties of the algorithm. They identified some unsecured issues, commonly generated in such algorithms, and how to avoid them.

Chengqing Li et al. [26] reevaluated the image scrambling encryption algorithm security. They stated that the internal correlation remaining in the cipher image disclosed corresponding information about the plain image. Finally, they concluded that the scrambling elements could be used to support plain text attacks. Chunhu Li et al. [27] presented an image encryption algorithm depending on the three-dimensional (3D) chaotic logistic map. A chaos-based key stream was generated through a modified 3D chaotic logistic map. The proposed encryption scheme included diffusion and confusion properties. Several security tests were applied to measure the performance of the proposed scheme in measuring the cryptographic application suitability.

## 3. Parameter Hopped 3D Chaotic Map Image Encryption Scheme

**Figure 1:**
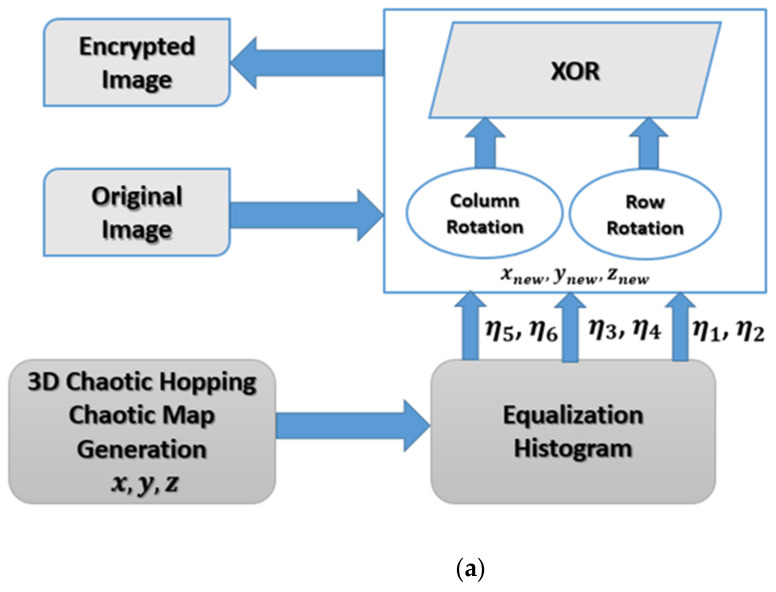

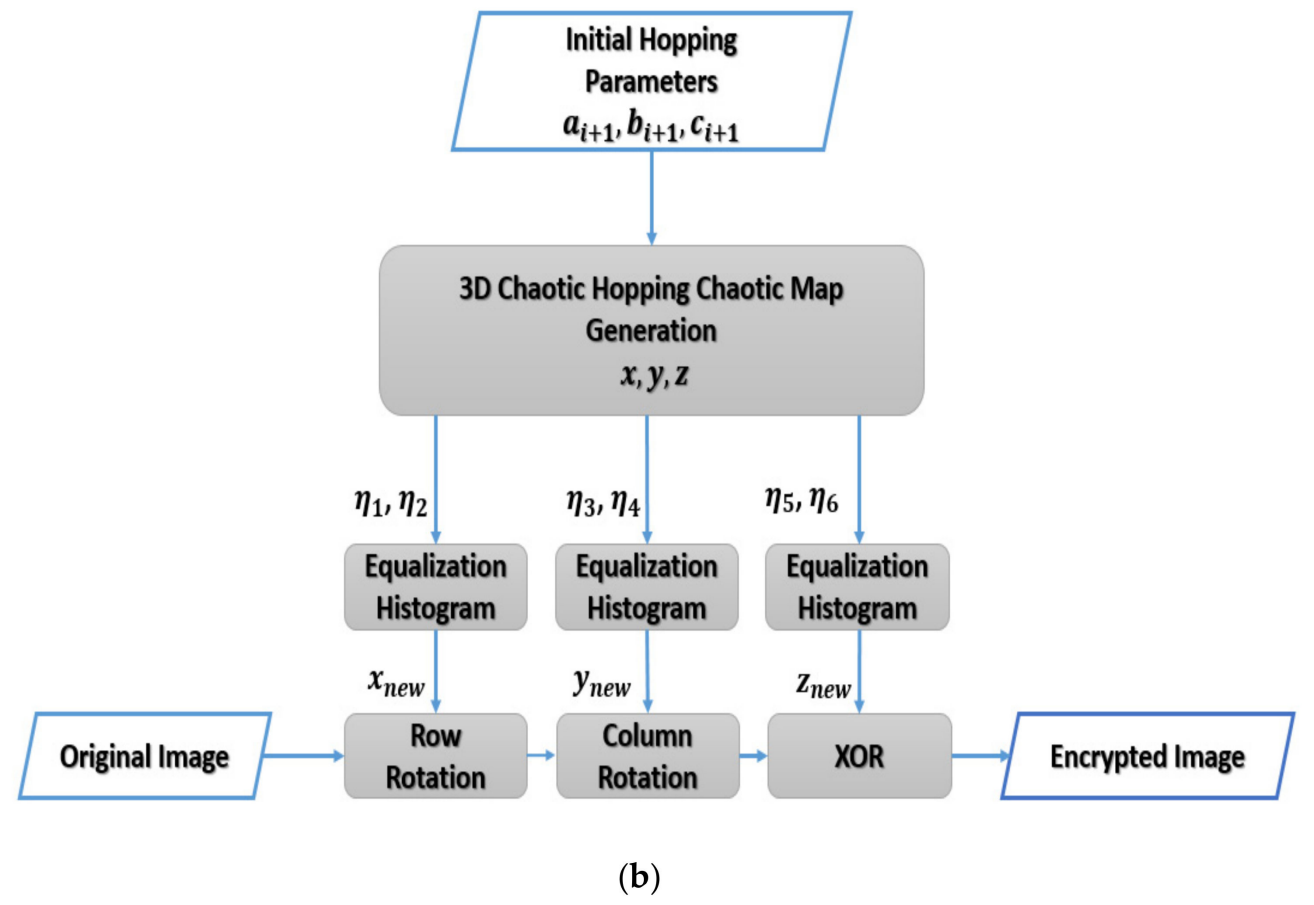
(**a**) Parameter hopped 3D chaotic map for image encryption scheme. (**b**) Flowchart of the proposed algorithm.

The proposed image encryption scheme is shown in Figure 1a and based on the parameter hopped 3D chaotic map. The image encryption scheme is generated through five main steps, namely parameter hopped 3D chaotic map generation, histogram equalization, row rotation, column rotation, and exclusive-OR (XOR) logic operation. Figure 1b represents the flowchart of the proposed algorithm.

### 3.1. Logistic Parameter Hopped 3D Chaotic Map Generation

#### 3.1.1. Generation of Initial Conditions

In this section, we describe our proposed algorithm to generate a pseudorandom bit sequence based on a logistic parameter hopping 3D chaotic map. The varying parameters for the 3D hopping chaotic map are $a_{i},b_{i}$ and $c_{i}$, and are generated through (1)–(4) under the specified initial conditions.

(1) $$a_{i+1}=a_{\max}-k_{i}\left(a_{\max}-a_{\min}\right)$$

(2) $$b_{i+1}=b_{\max}-k_{i}\left(b_{\max}-b_{\min}\right)$$

(3) $$c_{i+1}=c_{\max}-k_{i}\left(c_{\max}-c_{\min}\right)$$

(4) $$k_{i+1}=h_{0}\;k_{i}\left(1-k_{i}\right),\;\;\;\;\;for\;i=1,2,3,\ldots .$$

where $h_{0}=4$ and $k_{1}=0.01$ are the condition to make this equation chaotic. Here the above equations exhibit the chaotic behavior for $\;3.53<a_{i+1}<3.81$, $0.0001<b_{i+1}<0.022$ and $\;0.0001<c_{i+1}<0.015$ with initial values of $a_{1}=3.7900\;,\;b_{1}=0.0185\;,c_{1}=0.0125$ and its maximum and minimum values with $a_{\max}=3.81-0.0001\;$, $a_{\min}=3.53+0.0001\;$, $b_{\max}=0.022$, $b_{\min}=0.0001$ $c_{\max}=0.015$ and $c_{\min}=0.0001$.

#### 3.1.2. Generation of 3D Parameter Hopping Logistic Map

The 3D parameter hopping logistic map is generated through (5)–(7) as follows [28]:

(5) $$x_{i+1}=a_{i+1}\;x_{i}\left(1-x_{i}\right)+b_{i+1}\;y_{i}{}^{2}x_{i}+c_{i+1}z_{i}{}^{3}$$

(6) $$y_{i+1}=a_{i+1}\;y_{i}\left(1-y_{i}\right)+b_{i+1}\;z_{i}{}^{2}y_{i}+c_{i+1}x_{i}{}^{3}$$

(7) $$z_{i+1}=a_{i+1}\;z_{i}\left(1-z_{i}\right)+b_{i+1}\;x_{i}{}^{2}z_{i}+c_{i+1}y_{i}{}^{3}$$

where $a_{1}=3.7900\;,\;b_{1}=0.0185\;,c_{1}=0.0125\;,$ $x_{{}_{1}}=0.2350$, $y_{{}_{1}}=0.3500$, and $z_{{}_{1}}=0.7350$.

**Figure 2:**
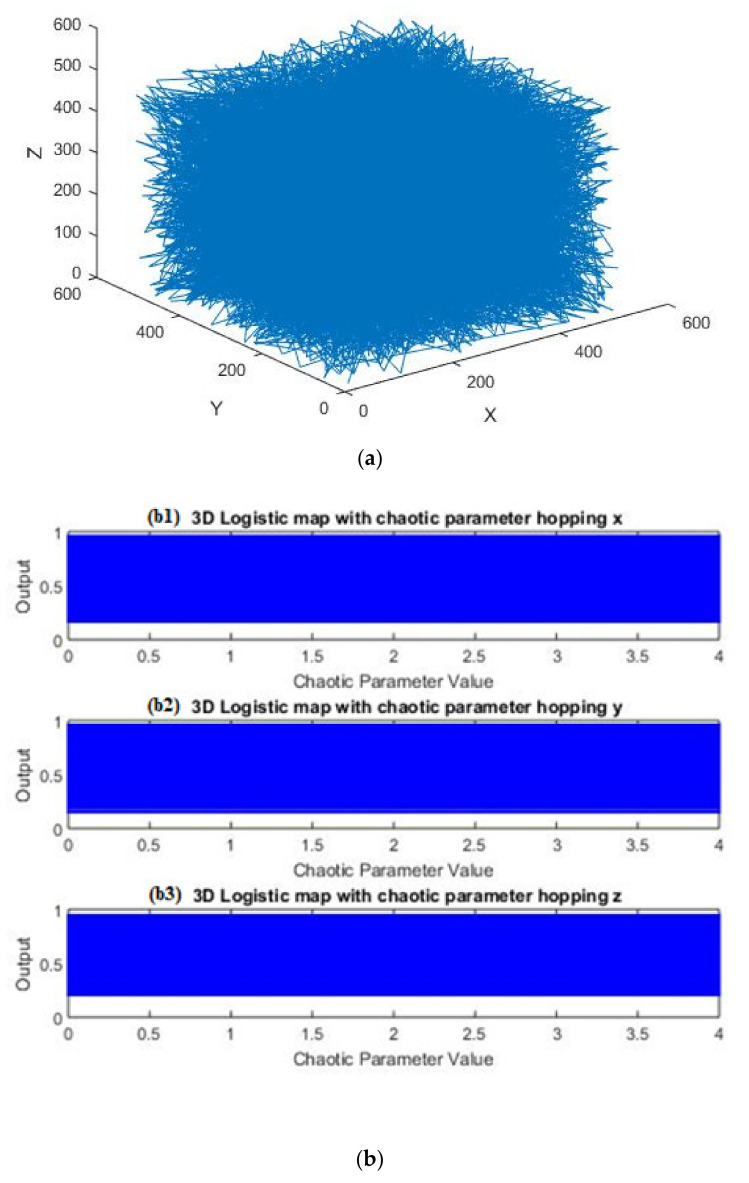
Chaotic Test: (**a**) chaotic behavior; (**b**) bifurcation diagram of hopped chaotic parameters *x, y* and *z*.

Figure 2a shows the chaos phenomena of the 3D parameter hopping logistic map depending on the varying parameters $a_{i},b_{i}$ and $c_{i}$ of the 3D hopping chaotic map. Figure 2b displays the bifurcation diagram of the 3D hopping parameters *x, y* and *z* obtained from Equations (5)–(7) with initial values of $a_{1}=3.7900\;,\;b_{1}=0.0185\;,c_{1}=0.0125\;,$ $x_{{}_{1}}=0.2350$, $y_{{}_{1}}=0.3500$ and $z_{{}_{1}}=0.7350$. It is clear that the bifurcation diagram of the proposed chaotic map has an enhancement in the parameter range of hopped chaotic sequence compared with the fixed chaotic parameters used in [28].

**Figure 3:**
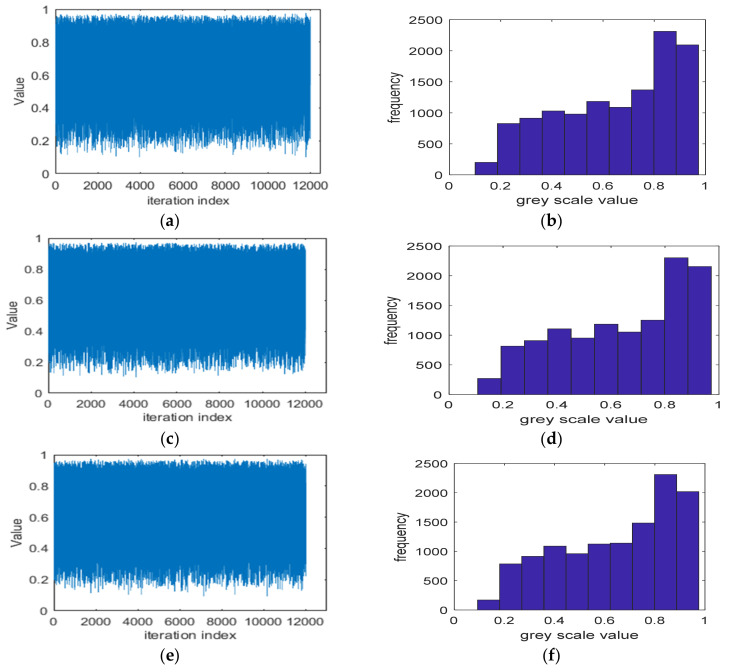
Values *x*, *y* and *z* and histogram generation. (**a**) Generated value of *x*, (**b**) histogram of *x*, (**c**) generated value of y, (**d**) histogram of *y*, (**e**) generated value of *z*, and (**f**) histogram of *z*.

The generated values and histogram generation of hopped chaotic sequence *x*, *y* and *z* obtained through (1)–(7) are depicted in Figure 3. Figure 3a,c,e shows the generated values for *x, y* and *z* with initial values of $a_{1}=3.7900\;,\;b_{1}=0.0185\;,c_{1}=0.0125\;,$ $x_{{}_{1}}=0.2350$, $y_{{}_{1}}=0.3500$ and $z_{{}_{1}}=0.7350$, while, Figure 3b,d,f represents the histogram for each obtained value of *x*, *y*, and *z*, respectively. Obviously, the histogram of the generated chaotic sequence has non-uniform distribution that may have an effect on the security of the system.

### 3.2. Histogram Equalization

**Figure 4:**
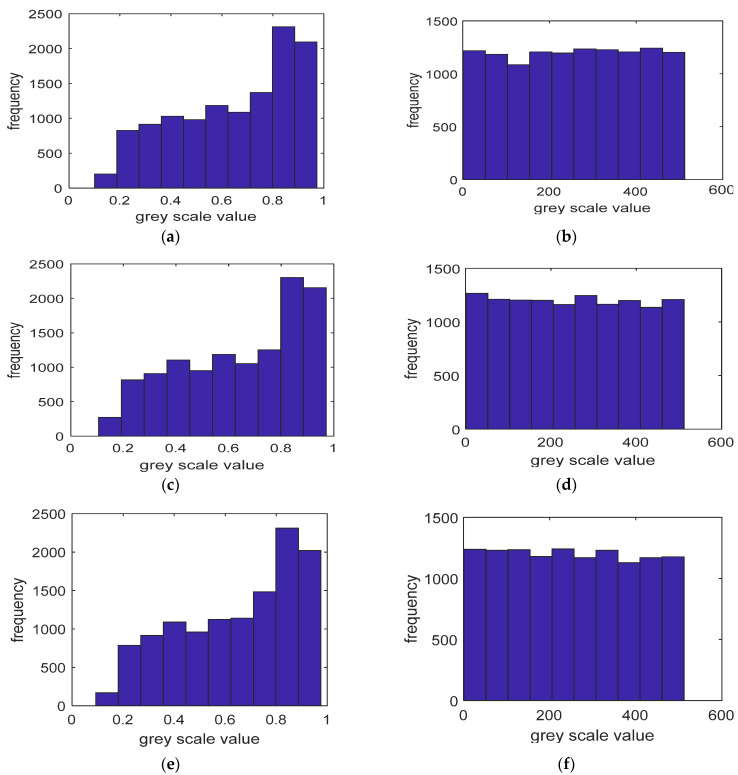
Histogram generation and equalization. (**a**) Histogram of *x*, (**b**) histogram equalized *x*, (**c**) histogram of *y*, (**d**) histogram equalized *y*, (**e**) histogram of *z*, and (**f**) histogram equalized of *z.*

The generated histograms displayed in Figure 3 are non-uniformly distributed. To further increase the security of the generated histograms, we apply an equalization process for x, y, and z through (8)–(10) as follows where $\eta_{2},\eta_{4}$ and $\eta_{6}$ are large random numbers and they are chosen to be equal and greater than 100,000 for simplicity, while *M* and *N* are chosen to be equal to the image dimension (256 × 256). It is clear from Figure 4b,d,f that after applying the above constraints, we obtain the equalized histogram for $x_{new},y_{new}$ and $z_{new}$.

(8) $$x_{new}=\left(\mathrm{integer}\left(x\times \eta_{2}\right)\right)\mathrm{mod}N$$

(9) $$y_{new}=\left(\mathrm{integer}\left(y\times \eta_{4}\right)\right)\mathrm{mod}M$$

(10) $$z_{new}=\left(\mathrm{integer}\left(z\times \eta_{6}\right)\right)\mathrm{mod}256$$

### 3.3. Row Rotation

For a gray image of M×N dimensions, the row rotation is executed by applying an offset value $\eta_{1}$, then selecting M elements of chaos sequence *x* beginning from the offset value $\eta_{1}$, and finally applying the chaos value *x* obtained through Equation (5) to rotate the row. To increase the security of the generated sequence, the row rotation could be right or left rotation according to the chaos value (odd or even).

### 3.4. Column Rotation

The column rotation is similar to the row rotation and can be applied by selecting N elements of chaos sequence *y*, choosing $\eta_{3}$ to be an offset value, then starting from $\eta_{3}$ and applying the chaos value *y* obtained from Equation (6). Now, we have an encrypted image with row and column rotation but with the same histogram of the original image. To overcome histogram attacks, we need to apply one more step to change the value of the image pixel as described in the following point.

### 3.5. XOR Operation

A final step in the encryption process is to XOR the generated sequence obtained through row and column rotations to get new pixel values other than the original ones. The XOR operation is done by converting the M×N image to a new 1 × MN image, then using an offset value $\eta_{5}$**,** XOR the chaos sequence *z* starting from $\eta_{5}$ and select M × N elements to finally get a well-secured encrypted image.

## 4. Statistical Tests Analysis and Simulation Results 

### 4.1. Simulation Setup

**Figure 5:**
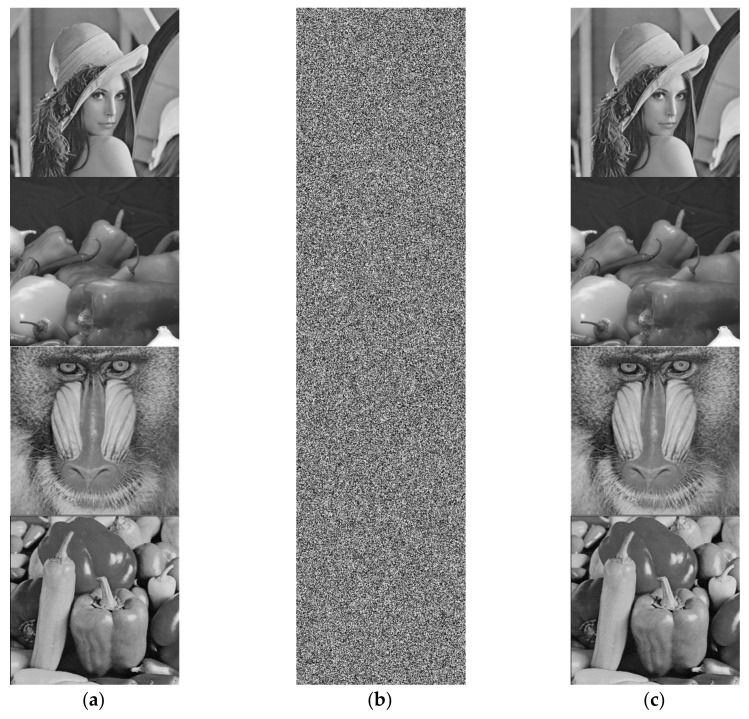
Simulation results for all images. (**a**) Original gray images, (**b**) encrypted gray images, and (**c**) recovered gray images.

The simulations were implemented in MATLAB R2015b (MathWorks, Natick, MA, USA) on a computer with Windows 10, Intel Duo Core I5 @2.53 GHz, 8 GB DDR3 RAM. The proposed cryptosystem was applied to a group of four gray images Lena, Deblur, Mandrill, and Peppers each with a dimension of 256 × 256 as shown in Figure 5a. The proposed 3D mapping encryption algorithm described in the previous section was applied by using the system parameters and initial values given in Table 1, which resulted in an encrypted version for the four selected images as shown in Figure 5b. Then we decrypted the cipher image to get the original image by using the correct key as shown in Figure 4c.

### 4.2. Statistical Analysis

**Figure 6:**
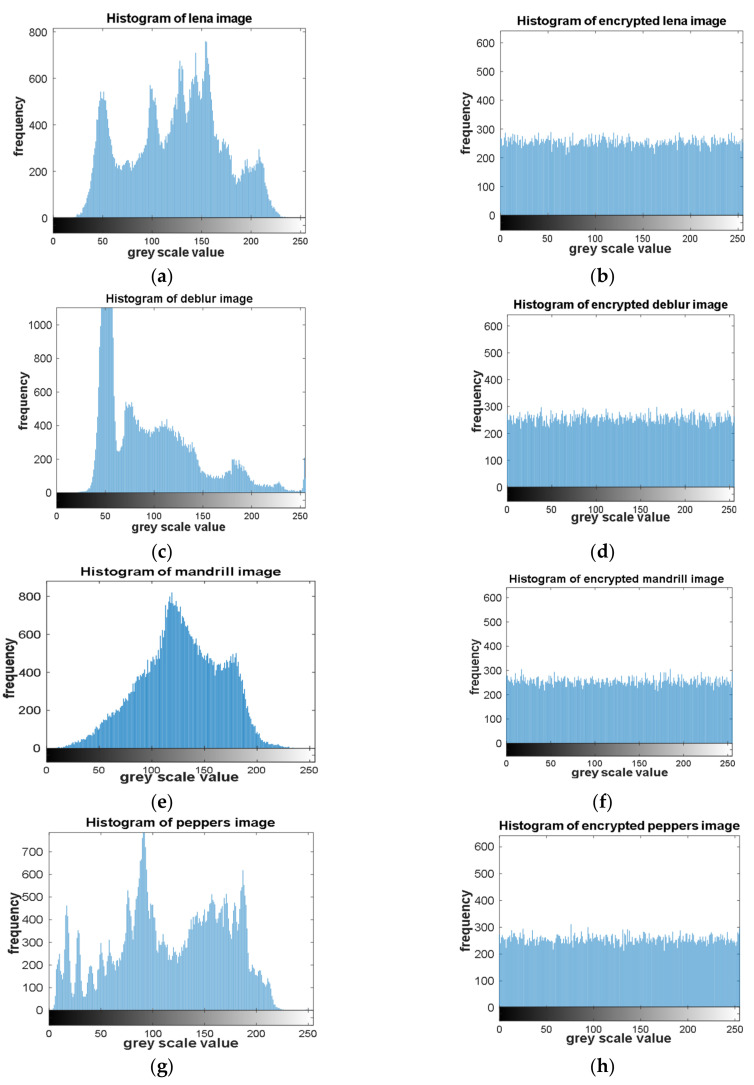
Histogram analysis for both original and encrypted images. (**a**,**c**,**e**,**g**) histogram of original images. (**b**,**d**,**f**,**h**) histogram of encrypted images.

Statistical attacks are a common type of image encryption attack due to the high correlation properties for adjacent pixels within an image. Such kinds of attacks could be avoided through randomly redistributing the pixels within the image and assigning a new value for each pixel. Figure 5 shows the histogram of the images under tests for both the original and encrypted versions. The encrypted images histograms are shown in Figure 6b,d,f and are uniformly distributed in terms of the pixel values compared to those in Figure 6a,c,e.Such uniformity distribution of the pixel values gives a good indication for the strength of the proposed encryption scheme.

### 4.3. Key Sensitivity Analysis

**Figure 7:**
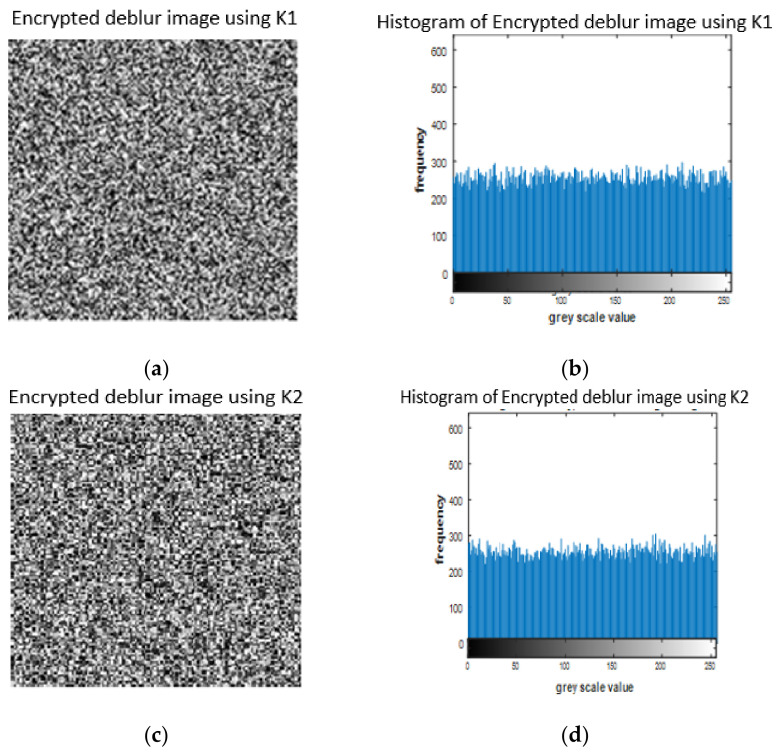
Key sensitivity analysis for deblur image. (**a**) Encrypted image using correct key. (**b**) Histogram of encrypted image using correct key. (**c**) Encrypted image using wrong key. (**d**) Histogram of encrypted image using wrong key.

Key sensitivity is a reliable test to measure the encryption cryptosystem strength for a digital image. The better the encryption algorithm, the more sensitive (against even a slight change in a single key) it should be. Table 2 depicts the parameters and initial values used to measure the key sensitivity of our proposed cryptosystem. Even with a variation in one bit in a single parameter between the encryption correct key (K1) and wrong key (K2) for the same image, we realized a difference in the resulting histogram obtained in both cases such as that shown in Figure 7.

### 4.4. NPCR and UACI Randomness Tests

Two of the most common tests used to measure the image encryption algorithm against differential attacks are NPCR and UACI. Mao and Chen [5,21], first introduced both randomness tests in 2004.

(11) $$\mathrm{NPCR}:F\left(C_{1},C_{2}\right)=\sum_{ij}\frac{D\left(i,j\right)}{M\times N}\times 100\%$$

(12) $$\mathrm{UACI}:U\left(C_{1},C_{2}\right)=\frac{1}{M\times N}\sum_{ij}\frac{\left|C_{1}\left(i,j\right)-C_{2}\left(i,j\right)\right|}{T}\times 100\%$$

(13) $$\mathrm{where},\;D\left(i,j\right)=\left\{\begin{array}{l} 0\;\;if\;C_{1}\left(i,j\right)\neq C_{2}\left(i,j\right)\\ 1\;\;\;if\;C_{1}\left(i,j\right)=C_{2}\left(i,j\right) \end{array}\right.$$

To measure the differential attacks, a randomly pixel of a plain image was chosen and a slight change in the pixel value occurred to get a new plain image. Then, the encryption algorithm was applied on both images to produce the cipher images C_1_ and C_2_ of the original and new images, respectively. NPCR and UACI are calculated and listed in Table 3. Sufficiently high NPCR/UACI values for both cipher images are usually considered as a strong resistance to differential attacks. The results depicted in Table 3 demonstrate that a slight variation in the original image caused no effect on the existing cryptosystem. However, a significantly larger difference was recognized for our proposed method, i.e., high sensitivity of the proposed cryptosystem even for a slight variation in the original image. The comparison of NPCR and UACI for the proposed and different algorithms on Lena image is demonstrated in Table 4.

### 4.5. Correlation Properties Analysis and Tests

The correlation values between two neighboring pixels in the original image was high and near to 1 for horizontal, vertical, and diagonal positions. Cryptanalysts usually exploit correlation to cause cipher break. To avoid such ciphered image attacks, adjacent pixels must be de-correlated, with low value and close to 0. The correlation formula is given by:

(14) $$corr\left(p,s\right)=\frac{\sum_{i=1}^{N}\left(p_{i}-\frac{1}{N}\sum_{j=1}^{N}p_{j}\right)\left(s_{i}-\frac{1}{N}\sum_{j=1}^{N}s_{j}\right)}{\sqrt{{\sum_{i=1}^{N}\left(p_{i}-\frac{1}{N}\sum_{j=1}^{N}p_{j}\right)}^{2}{\sum_{i=1}^{N}\left(s_{i}-\frac{1}{N}\sum_{j=1}^{N}s_{j}\right)}^{2}}}$$

**Figure 8:**
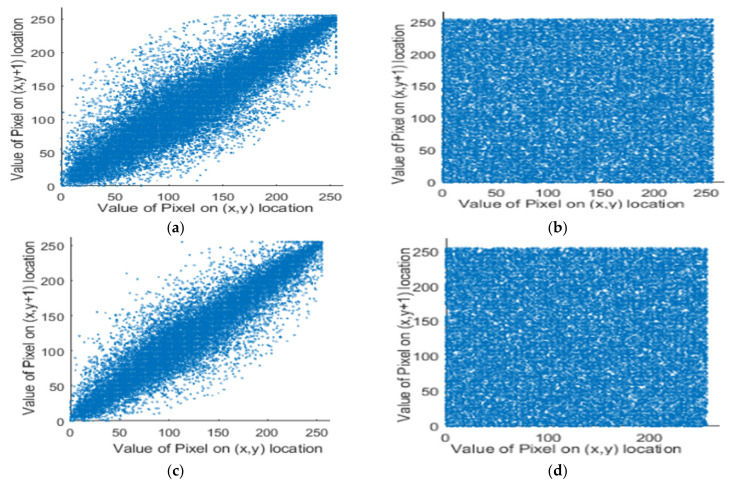
Correlation analysis of Lena image. (**a**) The vertical correlation, (**b**) vertical correlation for encrypted image, (**c**) horizontal correlation, and (**d**) horizontal correlation for encrypted image.

In Equation (14), *N* represents the total number of adjacent pixel and (*p_i_*, *s_i_*) are the adjacent pixels’ values. The correlation between two pixels for both original and ciphered images are depicted in Table 5, and Figure 8, respectively. Consequently, the proposed cryptosystem achieved zero-correlation and had a high privilege against correlation attacks. The comparison of correlation coefficient for the proposed algorithm and other algorithms for Lena image is demonstrated in Table 6.

### 4.6. Peak Signal-to-Noise Ratio (PSNR)

PSNR is defined by the quality estimator for image after compression or some modification like mean square error (MSE). Equations (15) and (16) represent the calculation of the PSNR and MSE respectively

(15) $$\mathrm{PSNR}=20\log_{10}\left(\frac{P_{\max}}{\sqrt{\mathrm{MSE}}}\right)$$

(16) $$\mathrm{MSE}=\frac{1}{M\times N}\sum_{i=1}^{M}\sum_{j=1}^{N}{\left(\left(C(i,j)-P(i,j)\right)\right)}^{2}$$

where, $P_{\max}$ is the highest pixel value of the gray image and its value is 255. P(i,j) and C(i,j) are the pixel value at a certain point (i,j) in the original image and the encrypted image, respectively. As long as the PSNR value is small, the resulted encryption algorithm will be more robust. The values of MSE and PSNR for the input tested images are listed in Table 7. The results of PSNR show that the proposed algorithm is very robust.

### 4.7. Noise Attack

**Figure 9:**
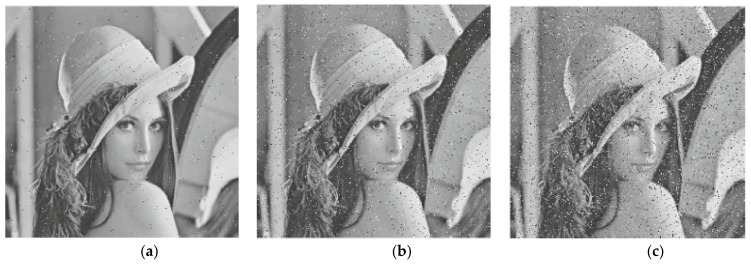
Salt and pepper noise attack results. (**a**) Intensity value 0.01, (**b**) Intensity value 0.05, (**c**) Intensity value 0.1.

During data transmission procedure, the opponent tries to decrypt the encrypted data. When the opponent fails to decrypt the ciphered data, he uses active or passive attacks to prevent the receiver from decrypting the encrypted data. Noise attack is one of most common ways used to distort the communication between the sender and receiver. Therefore, salt and pepper noise attacks were used with different intensity to measure the effect on the decrypted image. The results are provided in Figure 9. It may be visible that the proposed cryptosystem paper can be robust against the salt and pepper noise attack.

### 4.8. Entropy Analysis and Test Results

The entropy H of a message source S is obtained through the following formula:

(17) $$H\left(S\right)=-\sum_{i=0}^{N-1}P\left(S_{i}\right)\log_{2}P\left(S_{i}\right)$$

where *P (S_i_)* denotes the probability of (*Si).* Assuming the message source (*S)* emitting 256 pixel values of equal probability, the resulting entropy would be near to 8. The obtained entropy represents a truly random source and with an ideal value of the message source S. The uniform distribution indicates greater entropy information. An encrypted image with information entropy less than the ideal value would result in a high risk for the possibility of certainty, which means real image security is threatened. The obtained information entropy values through our proposed encryption scheme as seen in Table 8 refers to ideal values close to 8. The information entropy test results obtained for our proposed encryption scheme would give a good indication of the strength of the proposed algorithm against security threats.

### 4.9. Local Shannon Entropy

Local Shannon Entropy (LSE) is a new performance test to adjust the exact randomness by selecting the non-overlapping blocks inside the cipher image. This performance can be measured by computing the mean of the entropy analysis calculated in the previous section on each block in the cipher image. LSE can be expressed by

(18) $$H_{k,l}(S)=-\sum_{i=0}^{k}\frac{H(S_{i})}{k}$$

where, $S_{1},S_{2}\ldots \ldots S_{k}$ are particular k image blocks while l is the amount of pixels for each block. Table 9 illustrates the LSE values for the cipher image.. The results show that the LSE value for the proposed algorithm is nearer to the optimum value (≈8). Therefore, the proposed cryptosystem has high randomness.

### 4.10. Time Efficiency

Time efficiency is running on a computer with Windows 10, Intel Duo Core I5 @2.53 GHz, 8 GB DDR3 RAM (Dell, Round Rock, TX, USA). The time is calculated on both encryption and decryption process. The test is applied on proposed images of size 256 × 256 pixels. Table 10 records the time efficiency of the proposed system and different encryption schemes. The results show that the proposed algorithm is sufficiently fast compared with other schemes, and meets real-time performance necessities.

To summarize the performance analysis, Table 11 shows the analysis of the proposed algorithm compared with different schemes on the Lena image.

## 5. Conclusions

The main contribution described in this article is the proposal of a novel non–linear algorithm based on a logistic parameter hopped 3D chaotic map, using chaotic hopped parameters instead of fixed parameters for the chaotic map as well as the equalized histogram to increase the security of image encryption. First, dimensional permutation for the rows and columns of the image was obtained through our generated code. Secondly, we assigned the generated random values for the pixels during the value transformation stage. The steps required to build our encryption scheme involved starting with the code generation, followed by the position permutation and shuffling the rows and columns, then applying value transformation for the image pixels ending with the XOR operation. Most of the previous encryption techniques were depending on chaotic maps that used codebooks as a source for code generation. The modulated algorithm added more randomness and scattering for the generated code, which was very difficult to predict. The proposed encryption scheme was evaluated under several statistical tests such as: entropy analysis test, key sensitivity test, correlation properties, peak signal-to-noise ratio, noise attacks, and randomness tests including UACI and NPCR. The obtained test results were compared to similar encryption schemes based on 3D chaotic maps to evaluate the strength of our proposed scheme. The obtained results showed a significant improvement for the system security and resistance against different types of crypto analytical threats compared to other image encryption schemes based on similar algorithms.

## Figures and Tables

**Table 1:** Simulation and test parameters.

Offset Parameters	Value	Function
$\eta_{2}$, $\eta_{4}$, $\eta_{6}$	100,000	Histogram Equalization
$\eta_{1}$	500	Offset value for Row Rotation Vector
$\eta_{3}$	600	Offset value for Column Rotation Vector
$\eta_{5}$	700	Offset value for XOR Operation Vector

**Table 2:** List of the keys used for key sensitivity analysis.

Correct Key K1	Wrong Key K2
$x_{1}$= 0.2350	$x_{1}$= 0.2350$+10^{-17}$
$y_{1}$= 0.3500	$y_{1}$= 0.3500
$z_{1}$= 0.7350	$z_{1}$= 0.7350
$a_{1}=3.7900\;$	$a_{1}=3.7900\;$
$b_{1}=0.0185\;$	$b_{1}=0.0185\;$
$c_{1}=0.0125\;$	$c_{1}=0.0125\;$
$\eta_{2}=\eta_{4}=\eta_{6}=100,000$	$\eta_{2}=\eta_{4}=\eta_{6}=100,000$
$\eta_{1}$= 500	$\eta_{1}$= 500
$\eta_{3}$= 600	$\eta_{3}$= 600
$\eta_{5}$= 700	$\eta_{5}$ = 700

**Table 3:** Differential analysis for various test images.

Sensitivity Analysis	Images			
	Lena	Deblur	Peppers	Mandrill
NPCR (%)	99.6490	99.6231	99.5941	99.6063
UACI (%)	33.5965	33.4190	33.5651	33.4729

**Table 4:** Comparison of the plain image sensitivity analysis in the Lena image.

Sensitivity Analysis	Proposed	[29]	[30]	[31]	[32]
NPCR (%)	99.6490	0.996097	0.995964	0.996124	0.996107
UACI (%)	33.5965	0.334557	0.334762	0.334591	0.334436

**Table 5:** Correlation coefficient for various test images.

Image	Position		
	Horizontal	Vertical	Diagonals
Lena	0.9807	0.9626	0.9276
Encrypted Lena	0.0013	0.0022	0.0028
Deblur	0.9915	0.9946	0.9528
Encrypted Deblur	−0.0039	0.0035	−0.0020
Mandrill	0.9324	0.9379	0.9740
Encrypted Mandrill	0.0057	0.0059	−0.0034
Peppers	0.9541	0.9544	0.7066
Encrypted Peppers	0.0011	−0.0039	−0.0028

**Table 6:** Comparison of correlation coefficient for Lena image.

Schemes	Position		
	Horizontal	Vertical	Diagonals
Proposed	0.0013	0.0022	0.0028
[29]	0.0020	0.0035	0.0027
[30]	0.0068	−0.0054	0.0010
[31]	−0.0039	0.0035	−0.0020
[32]	−0.0047	0.0040	−0.0034

**Table 7:** MSE and PSNR for various test images.

Images	MSE	PSNR
Lena	9832.1	8.2043
Deblur	8213.8	8.9853
Mandrill	9910.5	8.1698
Peppers	7505.5	9.3770

**Table 8:** Entropy analysis for encrypted test images.

Entropy Test Results		
Image	Original	Encrypted
Lena	7.4498	7.9984
Deblur	7.4223	7.9890
Mandrill	7.4390	7.9887
Peppers	7.4300	7.9894

**Table 9:** Local Shannon Entropy.

Image	Proposed System	[29]	[30]	[31]	[32]
Lena	7.907462	7.902838	7.903975	7.904512	7.904671
Deblur	7.907321	7.903369	7.903520	7.902741	7.905962
Mandrill	7.908132	7.903750	7.903028	7.902728	7.906211
Peppers	7.909584	7.902970	7.903511	7.902972	7.906520

**Table 10:** Time Efficiency.

Algorithms	Proposed System	[29]	[30]	[31]	[32]
Time	0.15827	3.60724	2.65247	1.42729	0.88924

**Table 11:** Summarize of Performance Analysis.

Performance Analysis	Proposed System	[29]	[30]	[31]	[32]
NPCR (%)	99.6490	0.996097	0.995964	0.996124	0.996107
UACI (%)	33.5965	0.334557	0.334762	0.334591	0.334436
Horizontal	0.0013	0.0020	0.0068	−0.0039	−0.0047
Vertical	0.0022	0.0035	−0.0054	0.0035	0.0040
Diagonals	0.0028	0.0027	0.0010	−0.0020	−0.0034
Shannon Entropy	7.907462	7.902838	7.903975	7.904512	7.904671
Time	0.15827	3.60724	2.65247	1.42729	0.88924
